# Multiple viewpoint visualization of small scale flow phenomena: application to cavitation on an ultrasonic horn tip

**DOI:** 10.1016/j.ultsonch.2026.108071

**Published:** 2026-09-22

**Authors:** Jernej Ortar, Jure Zevnik, Matej Sečnik, Marko Hočevar, Matevž Dular

**Affiliations:** University of Ljubljana, Faculty of Mechanical Engineering, Askerceva 6, 1000 Ljubljana, SI, Slovenia

**Keywords:** Acoustic cavitation, Visualization, Multi-view imaging, Virtual highspeed camera, 3D visualization, 3D reconstruction

## Abstract

Accurate three-dimensional (3D) characterization of transient multiphase structures remains a persistent challenge, particularly in cavitating flows where vapor–gas interfaces evolve rapidly and exhibit pronounced 3D effects. This work presents a multiple viewpoint imaging system that delivers full 360° coverage of small scale multiphase phenomena using 30 synchronously triggered cameras and microsecond high power illumination. Two acquisition modes are implemented: a simultaneous snapshot mode providing instantaneous circumferential imaging, and a sequential mode emulating a virtual highspeed camera circling around the object of interest at effective frame rates above 6 × 10^4^ frames per second. Application to acoustic cavitation at a 2 mm ultrasonic horn tip reveals dominant 3D asymmetries that in many cases cannot be identified from single view measurements. The importance of multiple viewpoint approach to ultrasonically induced cavitation research is further emphasized by comparison of volumes determined from single and multiple viewpoint approaches. Preliminary reconstructions using shape from silhouette method demonstrate the suitability of the datasets for volumetric reconstruction. The system offers a robust platform for spatially resolved multi‑view visualization and provides a foundation for future quantitative 3D reconstruction and validation of cavitating flows simulations.

## Highlights

• 360° imaging of transient cavitation with 30 synchronized cameras.
• Multi-view analysis reveals strong 3D cavitation asymmetries.
• Shape-from-silhouette enables coarse 3D cavitation reconstruction.
• Virtual rotating high-speed imaging above 6 × 10^4^ fps.

## Graphical abstract

**Figure:**
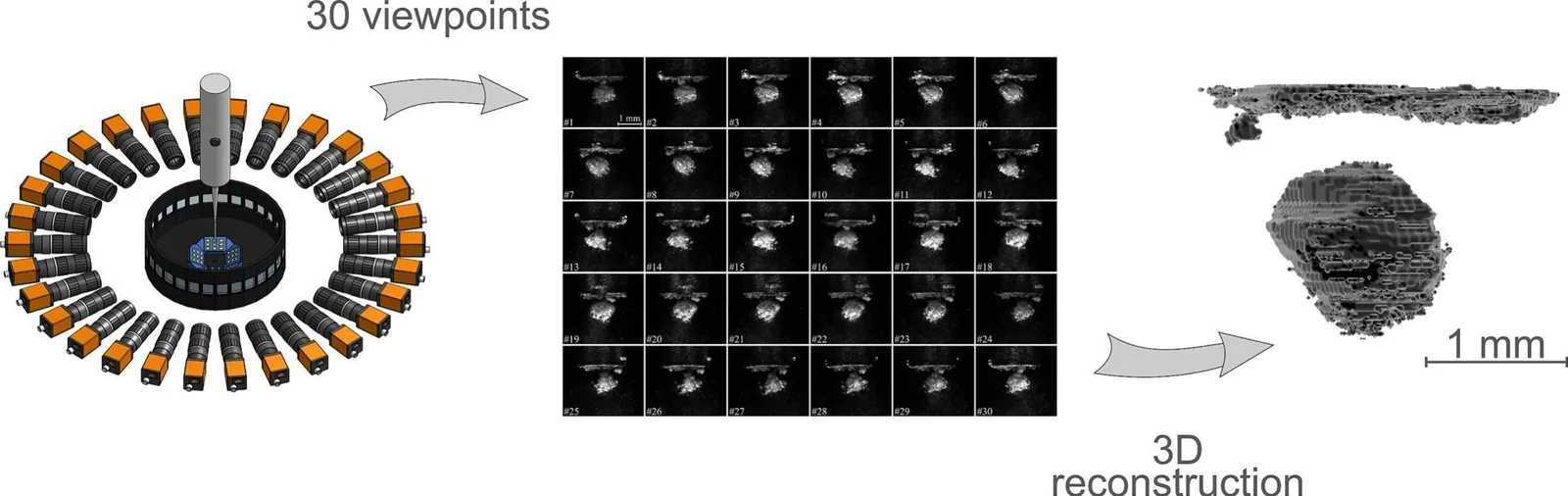

## 1 Introduction

Multiphase flow systems, including those involving cavitation, play an important role in modern engineering and are employed across numerous fields, ranging from chemistry, petrochemistry, and the food industry to nuclear technology [1], [2], [3]. A deeper understanding of these systems is therefore essential for improving existing technologies and enabling the development of new ones.

By their nature, multiphase flows are highly complex and contain a wide range of flow features, which makes them difficult to investigate, control, and monitor [4], [5]. One inherent characteristic of such flows is their three-dimensionality. Consequently, reducing their observations to two dimensions may lead to a loss of information and, in some cases, potentially misleading interpretations.

Experimental evaluation of cavitating flow structures is rarely performed in a manner that enables their local features to be fully distinguished. Techniques such as X-ray tomography are more commonly employed, allowing the phase boundary to be accurately determined within an observed cross section. To obtain a broader representation of the flow, individual cross sections are often time averaged and assembled into a quasi-spatially resolved representation [6], [7], [8]. However, this approach may result in the loss of important information regarding the instantaneous shape of the investigated phenomenon. Even partial 3D reconstruction methods remain relatively uncommon in the characterization of structures in twophase flows, including cavitation clouds, individual bubbles, and free surface topologies in open channel flows [9], [10], [11], [12], [13].

Earlier methods for obtaining 3D information on acoustic cavitation include highspeed holography and stereoscopic highspeed imaging. Lauterborn and Hentschel used highspeed holography to investigate the 3D dynamics of cavitation bubbles, while Appel et al. applied stereoscopic highspeed imaging to reconstruct spatial bubble structures and trajectories [14], [15]. These approaches demonstrate the value of 3D observations of cavitation phenomena, although they differ substantially from the simultaneous circumferential multiple viewpoint imaging approach employed in the present study. Despite these earlier 3D imaging efforts, only a limited number of studies have attempted volumetric reconstruction of cavitation structures from multiple viewpoints. These have primarily focused on cavitation developing on isolated propeller blade profiles. Pereira [16], [17], combined tomographic principles with stereo photography to estimate the volume and shape of cavitation clouds forming on a blade profile. Using four cameras, a silhouette based 3D shape reconstruction technique was applied to obtain a coarse volumetric description of the cavitation cloud. However, the setup was limited to four fixed perspectives, which inherently constrained the spatial resolution and completeness of the reconstructed shape [18]. A similar approach was later explored by Li et al. who investigated cavitation reconstruction on a blade profile using a two-camera stereovision system Their methodology, based on depth estimation from stereo image pairs, was described only briefly. Although such an approach enables partial 3D reconstruction, it remains inherently limited to the observed viewing direction, leaving substantial portions of the cavitation structure unresolved [18], [19]. A more advanced multiple viewpoint experimental setup was reported by Ebert and Damaschke [20], who employed 12 cameras arranged around a dedicated water tunnel. This configuration allowed tip vortex cavitation to be observed from twelve distinct perspectives. Although a full volumetric reconstruction was not achieved, the authors succeeded in mapping the spatial extent of cavitation on the blade surface. They suggested that such a system could provide a basis for future full 3D reconstructions and argued that complementing optical measurements with pressure sensors could support the development of cavitation detection and prediction models.

Preliminary investigations of stereo reconstruction based on silhouette extraction and stereo triangulation were performed by Savio et al. [21] for cavitation on a propeller. A similar experiment with an upgraded camera setup was later carried out by Franzosi et al. [22] for cavitation on a NACA 0015 hydrofoil. Yan [23] described the implementation of stereo reconstruction and triangulation algorithms, including an outline of the computational workflow applied to cavitation reconstruction. Both Yan and Savio et al. reported key experimental challenges associated with 3D cavitation reconstruction, including illumination control and specular reflections from vapor–liquid interfaces.

A further notable study was conducted by Shiraishi et al. [24], who investigated cavitation on a marine propeller. They employed a laser based triangulation technique using three line scan CCD cameras arranged in a single plane. This method enabled extraction of spatial information regarding the extent and volume of the cavitating region within the observation plane.

Beyond cavitation specific studies, several works have explored photogrammetric approaches for reconstructing bubble distributions in general twophase flows. These studies aimed to determine individual bubble sizes and their 3D spatial arrangement within bubble clouds [25], [26], [27]. Reported methodologies include combinations of particle image velocimetry (PIV) with stereography [28], as well as stereographic techniques employing colour filters to separate multiple viewing angles [29]. An especially innovative approach was proposed by Wang et al. [30], [31], who replaced multi-camera system with a single camera combined with multiple mirrors, thereby reducing hardware complexity and overall system cost.

Despite these efforts, accurate determination and interpretation of the 3D shapes of multiphase flow structures remain challenging. However, the shape of individual phases in a multiphase flow can be crucial for understanding their effects on the system in which they occur [32]. For example, in shedding cavitating flow through a Venturi restriction, the topology of the detached cavitation cloud has been shown to have a significant influence on cavitation erosion [33]. Another relevant example is cavitation generated at the tip of an ultrasonic horn. Although this phenomenon has been widely studied, its 3D nature has often been neglected [34], [35], [36], [37].

While the geometry of ultrasonic horns is typically axisymmetric, the gas–vapor structures forming at their tips are usually not. Fig. 1 presents two simultaneous views of a typical cavitation structure generated at the tip of an ultrasonic horn. This illustrates the limitations of classifying and geometrically describing such structures based on single viewpoint visualization [38], [39], [40].

**Fig. 1:**
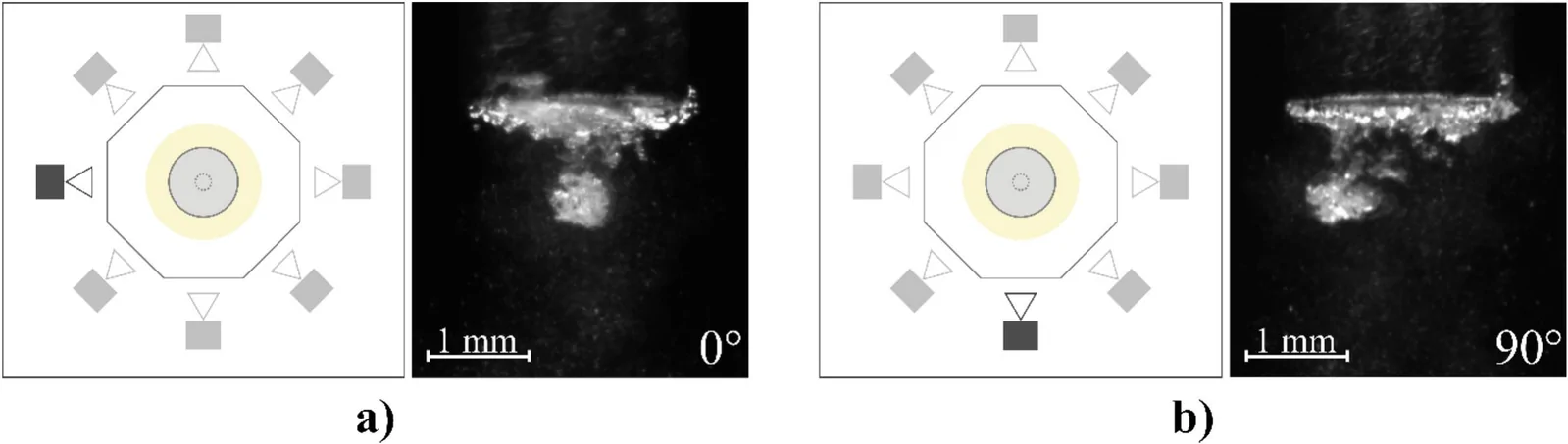
Typical acoustic cavitation cloud at the tip of an ultrasonic horn observed from two different viewing angles, revealing its complex 3D structure despite its nearly axisymmetric appearance from the 0° viewpoint.

In Fig. 1a, the structure appears as a uniform, axisymmetric cavitation cloud. However, Fig. 1b shows the same structure viewed from a direction rotated by 90° relative to Fig. 1a. From this perspective, the apparent “mushroom shaped cloud” is revealed to be a circumstantial projection of multiple detached cavitation clouds and structures. The example shown in Fig. 1 therefore highlights the importance of multiple viewpoint visualization for improving the interpretation of the geometrical complexity of multiphase phenomena such as cavitation.

Beyond the purpose of small scale flow phenomena observation, the output data obtained from the multiple viewpoint visualization system could provide a baseline for validation of 3D numerical simulations of multiphase flows (CFD), which are often validated using experimental data acquired through single point of view imaging [34], [37], [41].

In this paper we present and discuss a multiple viewpoint visualization system that enables circumferential observation of complex multiphase structures. Cavitation provides a particularly demanding benchmark, as it changes rapidly in time and space and is therefore difficult to observe without highspeed imaging. In many previous studies, limited camera numbers and restricted viewing coverage resulted in only coarse or partial descriptions of cavitation morphology, preventing the observation of important details within the cavitation cloud. In contrast, the present work adopts an extensive 30-camera arrangement, which substantially increases viewpoint coverage and enables a more complete and informative spatial characterization and observation of the cavitation cloud.

The remainder of the paper is organized as follows. Section 2 describes the experimental setup, including camera alignment, illumination, the synchronization subsystem, and acoustic cavitation generation. Section 3 presents the image acquisition procedure and the main system functionalities, namely the two principal acquisition modes. Section 4 presents preliminary results demonstrating improvements in volume estimation compared to single view approaches, as well as the potential of the system for further development toward quantitative small scale 3D reconstruction platform. Section 5 presents and discusses the experimental results, while Section 6 summarizes the main findings of the paper.

## 2 Experimental setup

The general purpose of the multiple viewpoint visualization system was to acquire, qualify, and potentially quantify as much spatial information as possible about the investigated flow. Owing to its highly transient nature, with collapse frequencies on the order of 5 kHz, and the strong light reflection from the continuously evolving liquid–gas interface, the cavitation cloud was selected as a benchmark phenomenon for evaluating the successful realization of the system.

To meet the defined research objectives and technical requirements, this in house visualization system was developed, with 30 interconnected cameras forming its core. The system described below was adapted for the investigation of small scale phenomena, such as acoustic cavitation at the tip of a sonotrode with a diameter of 2 mm. It can operate in two modes: snapshot mode and virtual spinning highspeed camera mode, both of which are described in the following section. The conceptual design of the experimental setup was first developed virtually as a CAD model and was subsequently physically constructed and gradually improved through iterative experimentation.

### 2.1 Multiple viewpoint imaging setup

#### 2.1.1 Camera hardware

The visualization system consisted of 30 cameras (VisionDatum LEO2 5000S-24GM), each equipped with a 2/3″ CMOS monochrome global shutter sensor with a resolution of 2448 × 2048 pixels and a pixel size of 3.45 × 3.45 μm. The ability to acquire images with exposure times as short as 1 μs made the cameras suitable for observing fast multiphase flows while minimizing motion blur artefacts.

All cameras were fitted with identical 50 mm prime lenses (ZLKC VM5028MP10) and 14 mm extension tubes. This combination of focal length and extension enabled sufficient image magnification for qualitative observations of cavitation clouds at length scales as small as 2 mm, while still satisfying the geometrical constraints of the experimental rig. The aperture was manually adjusted according to the illumination conditions and then kept constant at a f-number of f/6.3 for all experiments. An important characteristic of the selected lenses is their low TV radial distortion, specified by the manufacturer as − 0.01%. Camera settings were configured via the manufacturer software, enabling adjustment of image acquisition parameters including exposure time, trigger mode, sensor analog gain, bit depth, and region of interest and others.

Although the target phenomenon was highly transient and is typically investigated using highspeed cameras, slower and more cost effective cameras were intentionally used in the present system. A common limitation of imaging at very high acquisition frame rates is the reduction of effective spatial resolution or field of view. Therefore, each camera was operated in single shot mode, with exposure times on the microsecond scale to minimize motion blur, while preserving the native full sensor resolution for each acquisition.

#### 2.1.2 Geometrical arrangement

The cameras were mounted on a rigid circular frame with a nominal radius of 210 mm, measured from the center of the sonotrode to the front element of each lens. The 30 cameras were arranged with a uniform angular spacing of 12°, forming a complete 360° ring around the observed object, as schematically shown in Fig. 2.

**Fig. 2:**
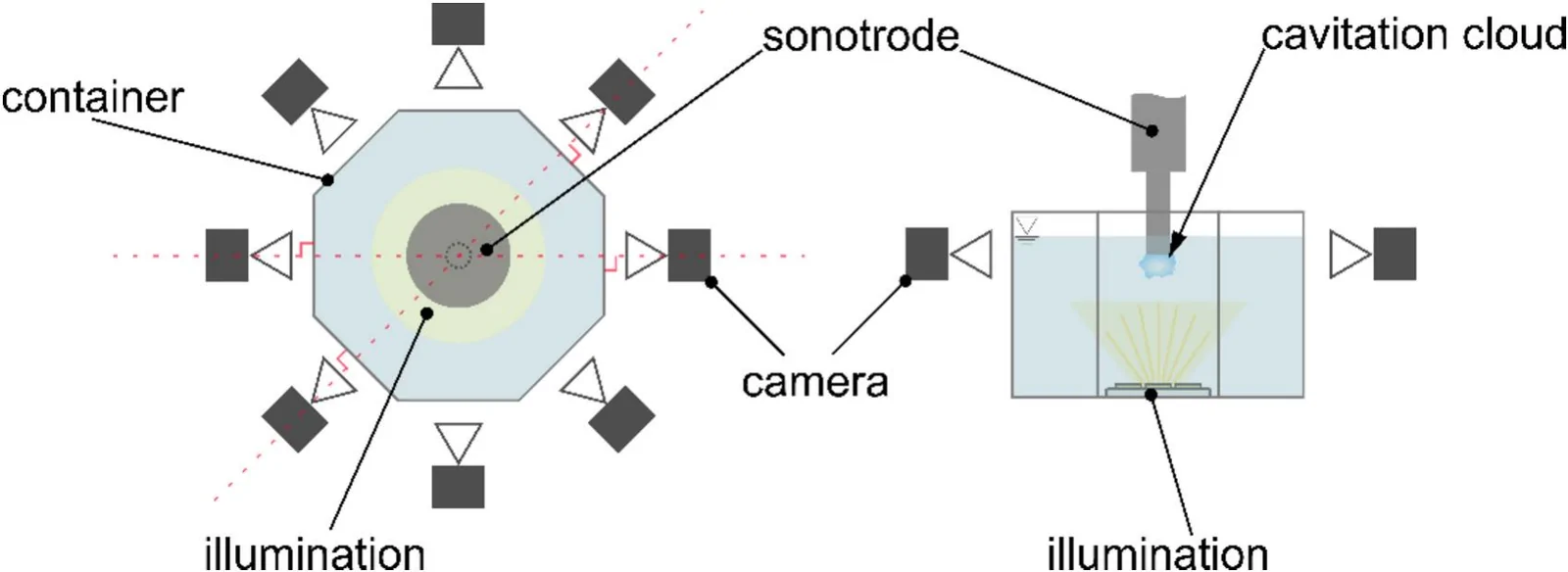
Schematics of experimental setup – top view (left) and side view (right). Only 8 cameras are shown for clarity.

The 12° angular spacing was selected to provide dense and regular multiple viewpoint sampling in the circular arrangement. With 30 cameras distributed over 360°, the configuration provides complete azimuthal coverage of the centrally located region of interest. This is particularly relevant for the subsequent shape from silhouette (SfS) reconstruction, where the reconstructed visual hull is constrained by silhouettes acquired from multiple viewing directions [42]. For the present rig radius of 210 mm, the 12° angular step corresponds to an adjacent view baseline of approximately 44 mm. All optical axes lay in a common plane and were directed toward the geometric center of the rig, providing a consistent viewpoint geometry.

The minimum working distance was dictated by the rig geometry and the physical dimensions of the optical components. At this distance, each camera provided sufficient field of view (FOV) and magnification for observing the cavitation cloud in the region surrounding the sonotrode tip. The optical magnification and field of view were determined by imaging an object of known dimensions and measuring its size in pixels. The measured magnification was M = 0.315, while the horizontal and vertical fields of view were FOVx = 26.8 and FOVy = 22.4 mm, respectively. At the full sensor resolution of 2448 × 2048 pixels, the measured field of view corresponds to an effective pixel size in object space of approximately 11 µm/pixel in both directions.

#### 2.1.3 Synchronization of cameras

The synchronization module architecture (Fig. 3) was developed around a single master trigger that controlled both the image acquisition timing of each camera and the onset of illumination. The master trigger signal was generated by a Teensy 4.0 microcontroller. Upon receiving a user applied trigger, the Teensy board sent a TTL-level trigger pulse either simultaneously to all cameras in snapshot mode or with a user defined delay in the virtual spinning highspeed camera mode. The propagation delays were measured to be well below the microsecond timescale and were therefore negligible relative to the 1 μs exposure time used to capture the rapidly changing cavitation structures. This level of synchronization was essential for producing a single, temporally frozen representation of the cavitation cloud shapes and structures. The same master trigger pulse was also forwarded to an Arduino Nano, which controlled the MOSFETs driving the LED illumination panels. The illumination was intentionally activated 100 μs before the camera trigger signals to ensure that the LEDs reached full intensity prior to image acquisition.

**Fig. 3:**
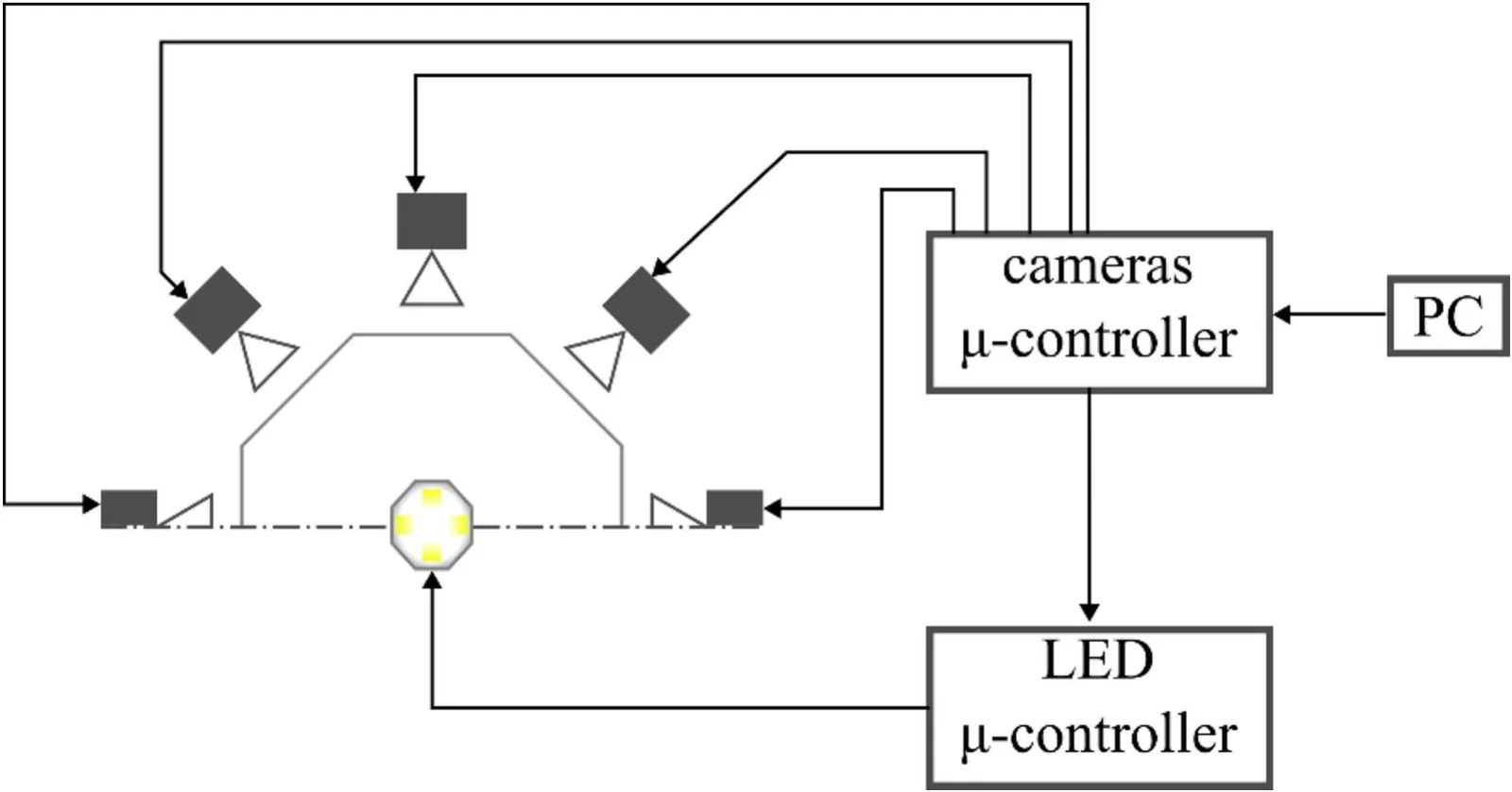
Schematics of camera and LED integration.

#### 2.1.4 Illumination

The illumination was provided by four custom built LED panels, each consisting of a 3 × 3 array of white LEDs (XINGLIGHT XL-C3570P8S11-62C3C2). The nominal maximum power dissipation of a single LED emitter was 19 W. The LED panels were positioned 20 mm below the sonotrode and oriented toward its tip at an angle of 35°. This illumination configuration was selected in an attempt to reveal as many structural details as possible on the surface of the cavitation cloud, while avoiding excessive backlighting and overexposure of the scene.

The LED assembly was powered by a 5 Ah LiPo battery pack controlled through the synchronization module. A 60C LiPo battery was selected because of its low internal resistance and its ability to deliver high currents over short time intervals. The maximum measured electrical power consumption of the illumination system was approximately 1 kW. The LEDs were pulsed for 300 μs to provide sufficient light intensity while preventing thermal damage, despite the short term overcurrent operation. Since the panels were submerged in water during operation, they were coated with a waterproof sealant.

### 2.2 Acoustic cavitation setup

The benchmark case selected for the present study was an acoustically generated cavitation cloud formed at the tip of an ultrasonic horn. The ultrasonic device used was a Hielscher UP200St, operated at a driving frequency of 26 kHz. The selected titanium sonotrode tip had a diameter of 2 mm. For the experiments described in this paper, the driving amplitude was set from 10% to 100%. The horn was submerged 30 mm below the free water surface, and the cavitation cloud region typically extended up to 3 mm from its tip. The ultrasonic transducer was fixed to an aluminium frame rack, ensuring that the sonotrode tip was consistently positioned at the geometric center of the camera rig.

The experiments were conducted inside an inhouse built, 30-sided container, as viewed from above, filled with deionized water. The container height was 80 mm, with each outer side measuring 22 mm in width. Optical access was provided through 1 mm thick cover glass windows with dimensions of 20 × 25 mm, mounted flush with the outer walls of the container. The total water volume used in the experiments was approximately 2.5 L. The final CAD model and the physical implementation of the system are shown in Fig. 4.

**Fig. 4:**
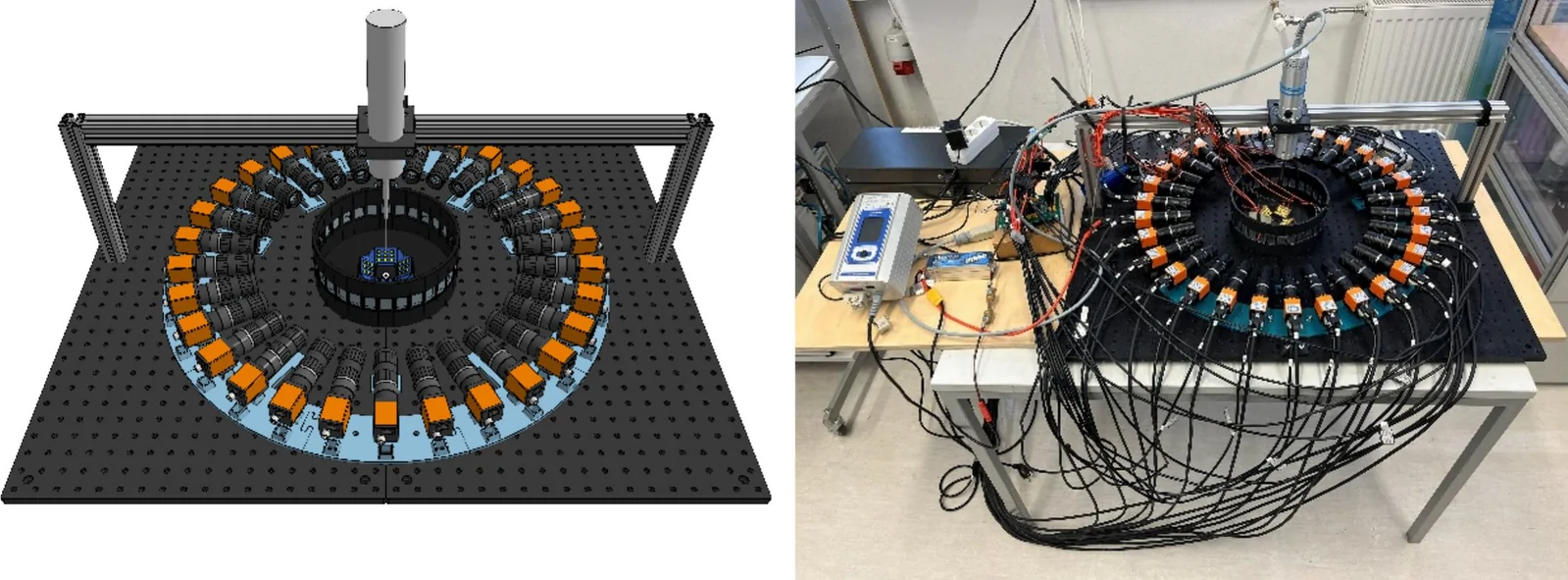
Conceptual CAD model of the multiple viewpoint visualization system (left) and its actual realization(right).

## 3 Image acquisition and processing

### 3.1 Acquisition modes and camera settings

Two acquisition strategies were used to characterize the cavitation structures surrounding the sonotrode tip.:

a) **A snapshot mode** in which all 30 cameras took an image simultaneously, and
b) **a virtual spinning highspeed camera mode** in which each camera was triggered with an arbitrary delay, creating the effect of a single highspeed camera rotating around the observed phenomenon at a constant velocity. Schematic representation of this mode operation is presented in Fig. 5.

**Fig. 5:**
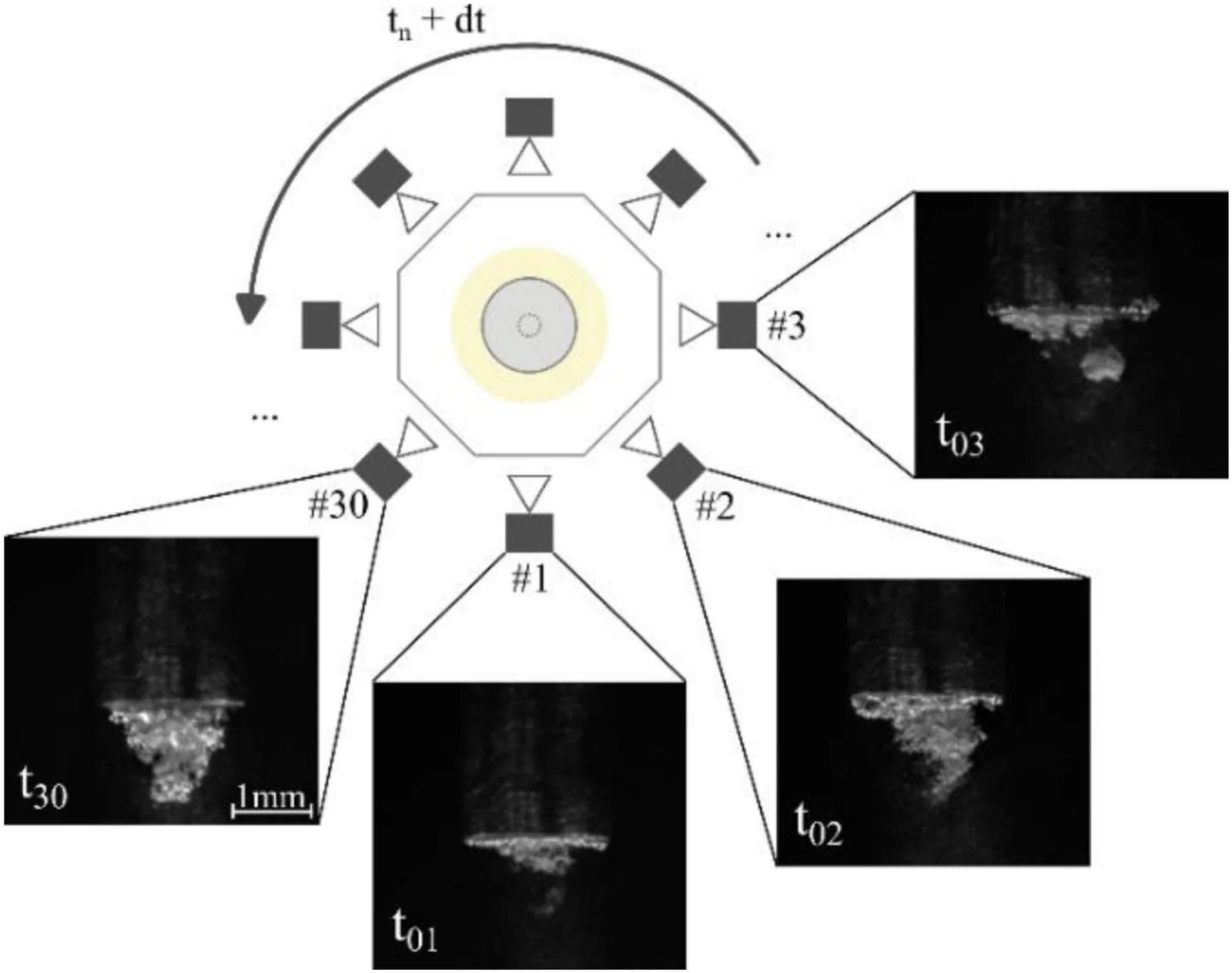
Schematic representation of the virtual spinning highspeed camera mode achieved through controlled time delays between successive camera acquisitions.

In *snapshot mode* all cameras acquired images simultaneously, providing multiple viewpoint snapshot of the observed phenomenon. This approach maximized spatial information at a single instant and provided suitable input data for 3D spatial reconstruction. However, because the acquisition was limited to a single time point, the method resolved the phenomenon primarily in the spatial domain rather than simultaneously in both space and time.

In *virtual spinning highspeed camera mode*, the cameras remained fixed but were triggered sequentially with a time delay Δt between successive acquisitions. This produced a circumferential image sequence over the total acquisition time, defined as

(1) $$\begin{array}{c} t_{tot}=\Delta t\cdot \left(N-1\right), \end{array}$$

where N is the total number of cameras. The resulting sequence approximated the view that would be obtained by a single highspeed camera rotating around the cavitation region. In this mode, the detailed instantaneous 3D structure was not captured. Instead, the method enabled partial visualization of the temporal development of characteristic cavitation patterns around the full circumference of the observed region. The effective frame rate was determined by the time delay between consecutive camera triggers and is given by

(2) $$\begin{array}{c} f=\frac{1}{\Delta t}. \end{array}$$

The minimum achievable Δt was limited by the camera exposure time and the synchronization system, allowing effective frame rates of up to f = 1 Mfps.

Both acquisition strategies used identical optical setups, synchronization electronics, camera arrays, and acquisition settings, and the only difference was the camera triggering sequence. In all experiments, the 30-camera system provided complete $360^{{}^{\circ}}$ coverage of the region surrounding the sonotrode tip. The recorded images consistently showed the sonotrode tip and a cavitation cloud extending up to approximately 3 mm from the tip.

### 3.2 Acquisition protocol

Each acquisition cycle followed the same sequence:

1. The ultrasonic horn was set to the desired operating state.
2. A stable cavitation regime was established.
3. The Teensy microcontroller issued the master trigger. (The acquisition was initiated manually, either by pressing a trigger button or by a software command, and was not synchronized with the sonotrode excitation or acoustic field.)
4. LED panels generated a 300 μs illumination pulse overlapping the exposure window.
5. Cameras initiated a synchronized 1 μs exposure. In snapshot mode, all cameras received the trigger simultaneously, whereas in virtual spinning highspeed camera mode triggers were issued sequentially according to user defined delays.
6. Images were read out, stored, and labelled according to camera ID and operating conditions.

A complete 30-camera acquisition generated approximately 143 MB of data, stored locally as 8-bit monochrome TIFF images. A minimum interval of 1 s was imposed between successive acquisitions as a safety measure to prevent overheating of the pulsed LED illumination system. Since shorter acquisition intervals were not investigated, the maximum acquisition rate permitted by camera readout, data transfer, and storage was not determined independently.

## 4 Proof of concept 3D reconstruction

In snapshot mode, in addition to providing simultaneous visualization of the cavitation cloud from multiple viewpoints for qualitative evaluation, the secondary objective was to acquire images suitable for reconstructing the cavitation cloud geometry and estimating its volume. This was intended as an improvement over conventional volume estimation approaches based on revolving a projection obtained from a single visualization view. Such capability was considered a useful addition to the system and a potential basis for quantitative evaluation of spatially resolved cavitation features. To demonstrate the ability of the system to acquire multiple viewpoint datasets suitable for 3D reconstruction, a simplified shape from silhouette reconstruction method [18], [43] was applied.

To simplify the reconstruction procedure, the camera model was reduced to an orthographic projection directed toward the centre of the experimental setup. Thus, instead of applying a full pinhole camera model [43], commonly used for entocentric lens, perspective induced changes in apparent object size were neglected. This approximation was considered acceptable because the reconstruction was used primarily to estimate the global 3D shape, rather than to obtain metrically exact surface geometry. Camera misalignment was corrected using reference images of the ultrasonic horn recorded at rest. The centre of the horn tip was detected by a simple edge detection procedure and used as a geometric reference point for image cropping and alignment between different viewpoints. The aligned silhouette masks were then used for voxel occupancy evaluation and subsequent reconstruction of the 3D object shape. The reconstruction volume was first defined as a regular voxel grid surrounding the region of interest. For each camera view, binary threshold masks were generated to represent the interior of the object silhouette. A voxel was retained if its projection fell inside the corresponding silhouette masks for all considered viewpoints, otherwise it was removed from the reconstruction volume. The resulting set of occupied voxels therefore approximated the visual hull of the observed object.

For verification and validation purposes, two preliminary tests were performed using a CAD model and a 3D printed solid object of the same geometry (Fig. 6). The first test used synthetic images of the truncated cube like CAD object with known geometry. The primary purpose of this test was to verify the simplified SfS reconstruction procedure by comparing reconstructions obtained using arbitrary number of viewpoints with the ground truth CAD geometry.

**Fig. 6:**
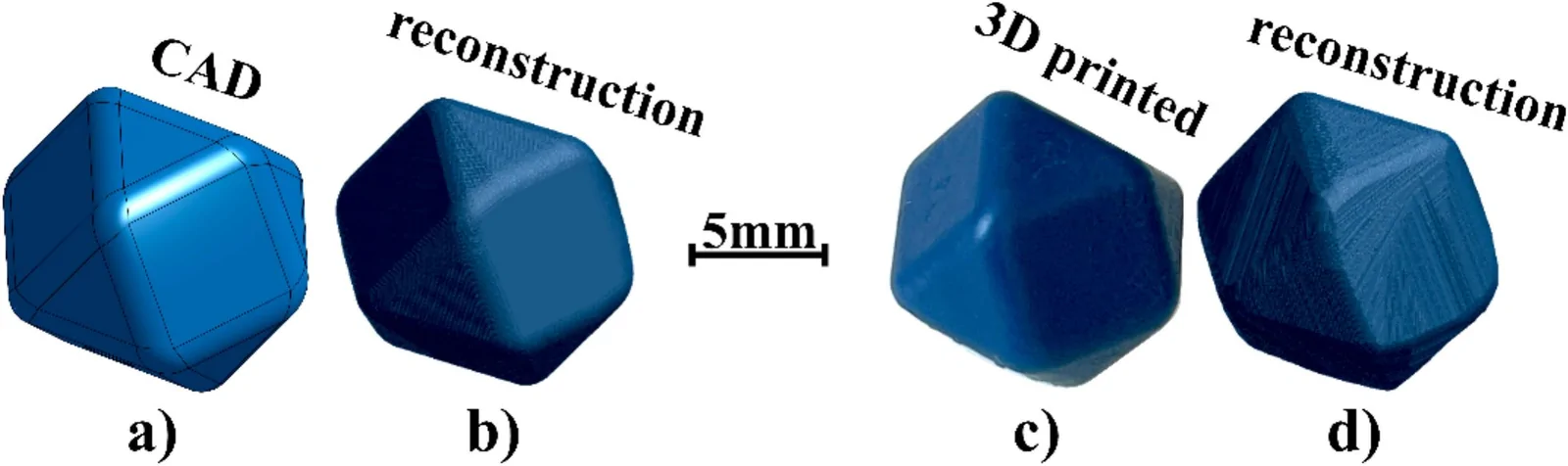
(a) Synthetic CAD model and (b) corresponding SfS reconstruction; (c) 3D printed test object and (d) corresponding SfS reconstruction. Animated sequences of test objects and their 3D reconstructions are provided as supplementary Videos S1 and S2.

The results presented in Fig. 7 were obtained from synthetically generated object views corresponding to the camera positions available in the multiple viewpoint visualization system. For method verification purposes, additional synthetic datasets with more than 30 viewpoints were included. Reconstructions were performed using different numbers of viewpoints, ranging from 3 to 360, with the latter corresponding to an angular spacing of 1°. The results show that the reconstructed volume error decreases as the number of viewpoints increases. The median volumetric relative error is 18.2% for 3 viewpoints, 8.89% for 10 viewpoints, 4.02% for 20 viewpoints, 2.38% for 30 viewpoints, and 0.05% for 360 viewpoints, indicating progressively improved volumetric accuracy with denser angular sampling. This confirms that, when applying the SfS method, the use of a larger number of cameras improves reconstruction accuracy, particularly for volume estimation of reconstructed objects.

**Fig. 7:**
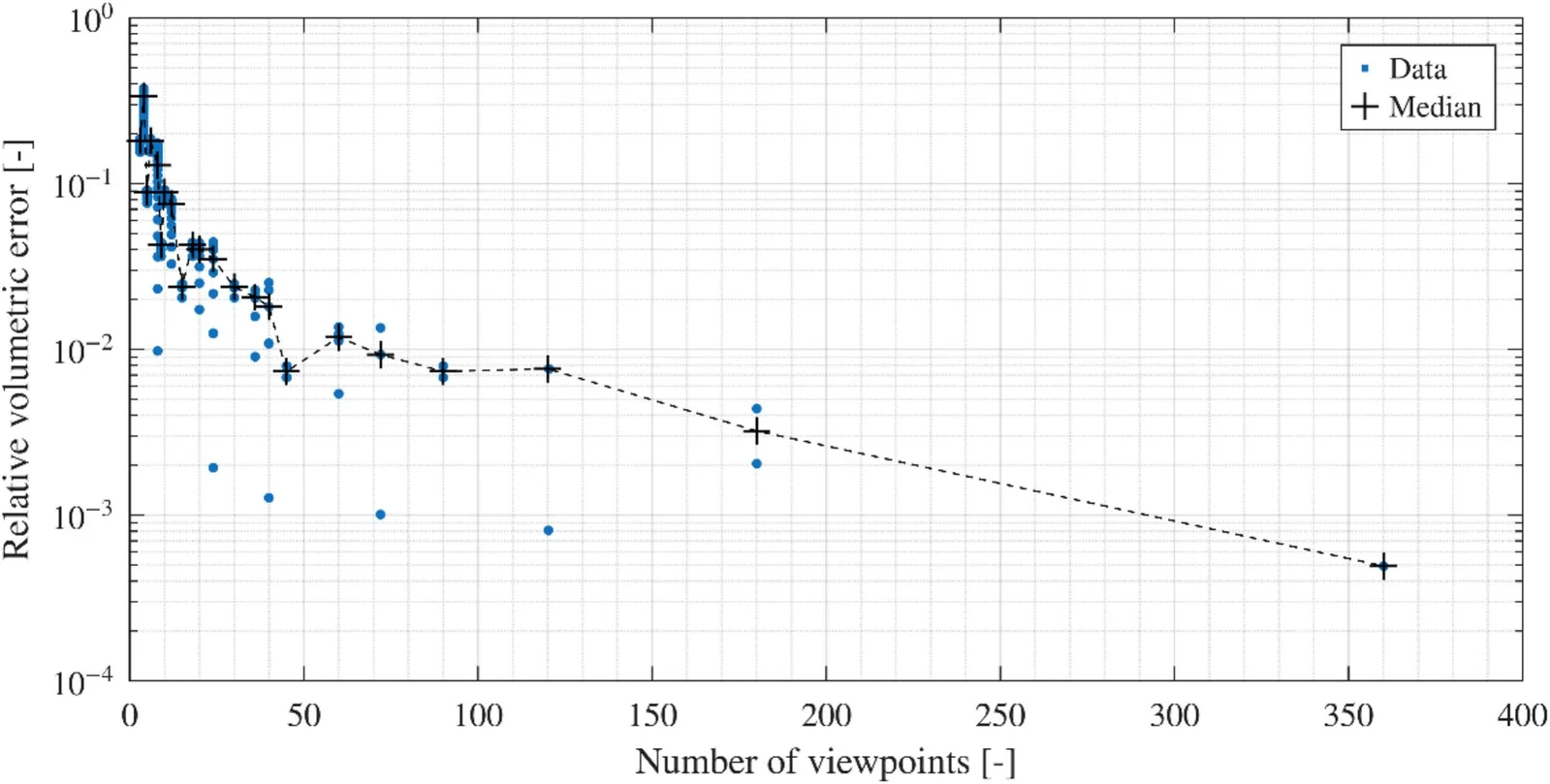
Relative volumetric errors with respect to the ground truth CAD model.

The second test was performed as a coarse estimation of the order of magnitude of the error associated with the current multiple viewpoint visualization system. For this purpose, the same CAD object was fabricated using SLA 3D printing technology (Fig. 6b) and subsequently reconstructed from images acquired by all 30 cameras of the visualization system. Different combinations of these 30 images, as presented in Fig. 8, were then selected for reconstruction to evaluate the influence of both the number and arrangement of viewpoints. For example, when three viewpoints were used, ten independent reconstructions were performed, each based on a different combination of three images. The reference volume of the 3D printed object was calculated from its measured physical dimensions rather than from the nominal CAD dimensions, thereby accounting for possible dimensional deviations introduced during manufacturing.

**Fig. 8:**
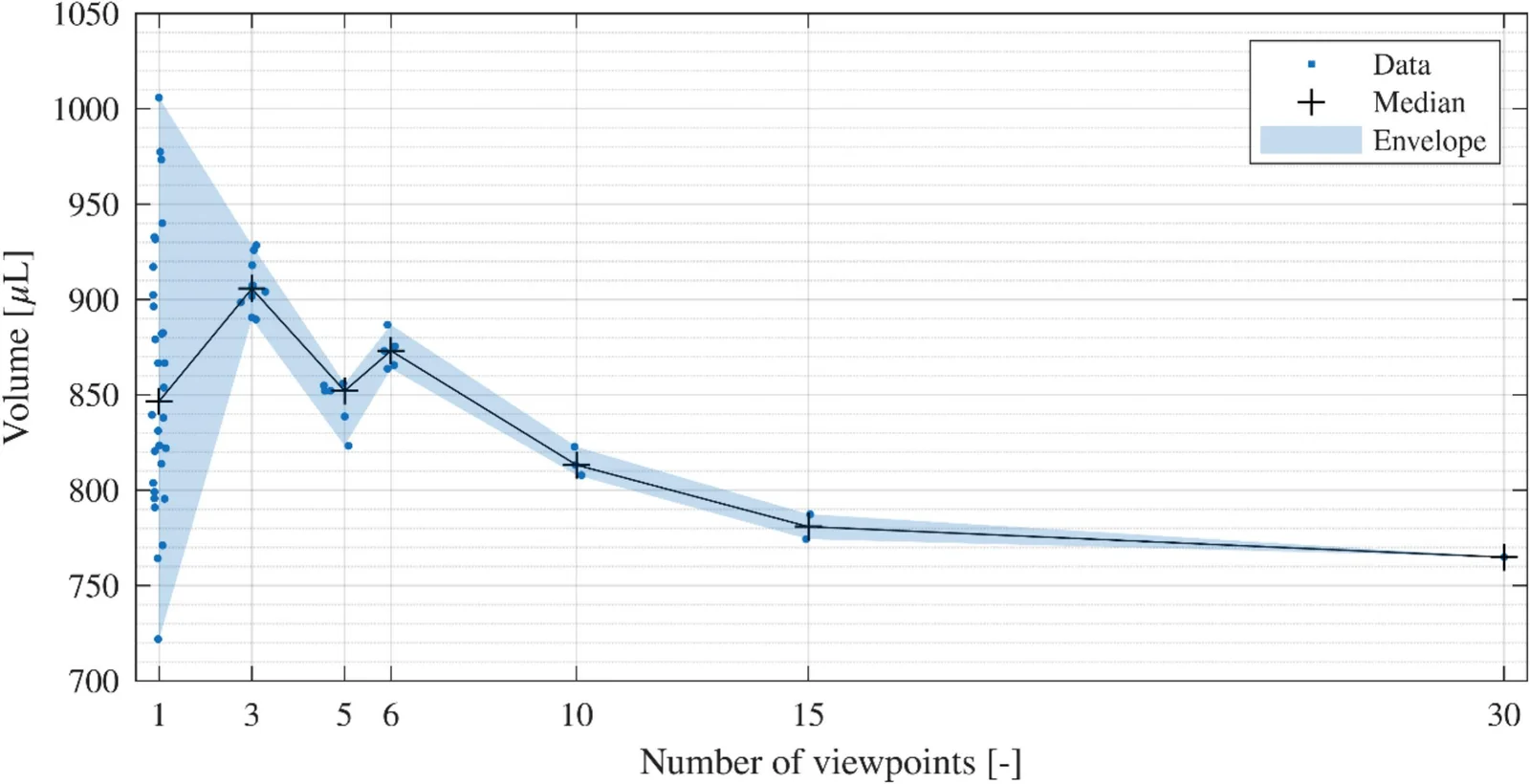
Volume estimates as a function of the number of cameras used in the SfS reconstruction procedure. Data points corresponding to a single camera were obtained using the volume of revolution approach. Individual observations are horizontally offset within each amplitude group to reduce marker overlap; the horizontal offset has no physical meaning.

The results of the second test are presented in Fig. 8. The variation in estimated volume decreases as the number of viewpoints used for reconstruction increases. Furthermore, from six viewpoints onward, the median values appear to converge asymptotically toward a limiting value with an increasing number of cameras. From the measured dimensions of the 3D printed object, one can estimate its volume and compare it to the one obtained by the SfS reconstruction using 30 viewpoints. The comparison yields a relative volumetric error of 10.7%. Although the volumetric discrepancy was 10.7%, volume scales with the third power of the characteristic linear dimensions. If this discrepancy were attributed to uniform scaling in all three spatial directions, it would correspond to an equivalent linear discrepancy of less than 3.7%. This can be considered promising, especially when the average discrepancy for cavitation cloud volume obtained by the axisymmetric volume of revolution assumption (current state of the art) is 172%, even as much as 600% in specific cases (see Fig. 12 and its discussion).

It is important to note that the data points corresponding to a single viewpoint represent volume estimates obtained by assuming solids of revolution from each individual viewpoint in the visualization system. Since the experimental images rarely exhibit perfectly symmetric silhouettes, the left and right halves of each silhouette were revolved independently through 180°, after which the resulting volumes were summed. The substantial scatter of these data points indicates a high degree of variability in the estimated volumes, demonstrating that the single view volume of revolution approach is strongly dependent on the observation angle. This dependence is already evident for a relatively regular test geometry, such as the truncated cube like object used here It is therefore expected to be even more pronounced for highly irregular and asymmetric structures, as was further confirmed by the cavitation experiments.

## 5 Results and discussion

### 5.1 Simultaneous image acquisition

In snapshot mode, the experimental design focused on simultaneous camera triggering to acquire time synchronized images of the same phenomenon. Individual images in Fig. 9 show 30 distinct viewpoints of the same instant of acoustic cavitation cycle at the 75% amplitude. Camera positions are labelled from #1 to #30, with numbering proceeding counterclockwise when viewed from above, as shown in Fig. 9.

**Fig. 9:**
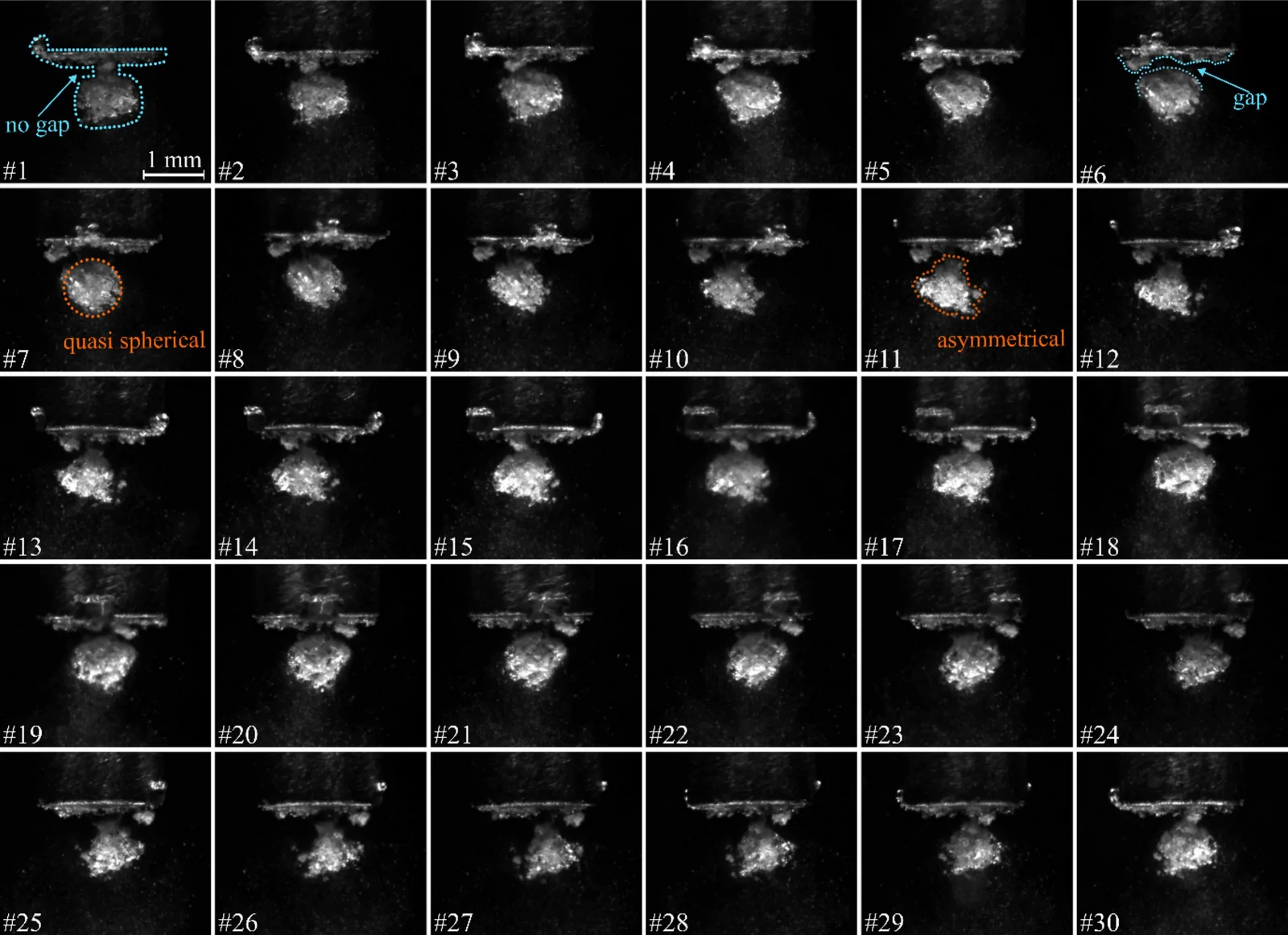
A set of 30 images recorded at the same instant by all cameras. Additional animated views are provided in supplementary Video S3.

The sequence in Fig. 9 reveals a wide range of cavitation structures and morphologies. Larger structures, such as the main cavitation cloud, are visible from all viewpoints, together with smaller individual bubbles dispersed in the surrounding water and extending from the sonotrode tip in a conical pattern.

Analysis of a detached region of the same cavitation cloud, frozen in time and observed from 30 different angles, reveals pronounced variations in its apparent position, shape, and silhouette projection. From camera position #1, the cavitation cloud appears attached to and centred beneath the ultrasonic horn tip, forming an apparently symmetric structure. However, when observed four positions further counterclockwise, corresponding to camera #6 and an angular displacement of 60°, a clear gap becomes visible between the horn tip and the largest cavitation structure. The feature that initially appears to form a continuous connection between the quasi-spherical vapor region and the horn tip can then be identified as a separate structure attached only to the edge of the tip. The apparent shift in the spatial position of the spherical cloud further demonstrates that structures which appear highly symmetric from one viewpoint can in fact be strongly asymmetric, both about their own vertical axis and with respect to the symmetry axis of the sonotrode tip.

This perspective dependent variability is also evident in the apparent shape of the largest cavitation cloud. Although it appears nearly spherical from many viewpoints, images acquired from positions #10, #11, and #12 reveal dimples and concavities that are not visible from other viewing angles. When observed from a certain single viewpoints, the cavitation cloud may appear as a uniform and coherent structure. A change in perspective, however, shows that this apparent uniformity is a projection artifact. The cloud is instead composed of several smaller substructures, whose overlap can create the visual impression of a single continuous volume.

Using the images presented in Fig. 9, 3D reconstruction was performed using the method described in Section 4. Five representative views of the reconstructed result are shown in Fig. 10 to provide a qualitative assessment of the reconstruction outcome. Animated sequences covering 360° field of view can be found in supplementary Video S3.

**Fig. 10:**
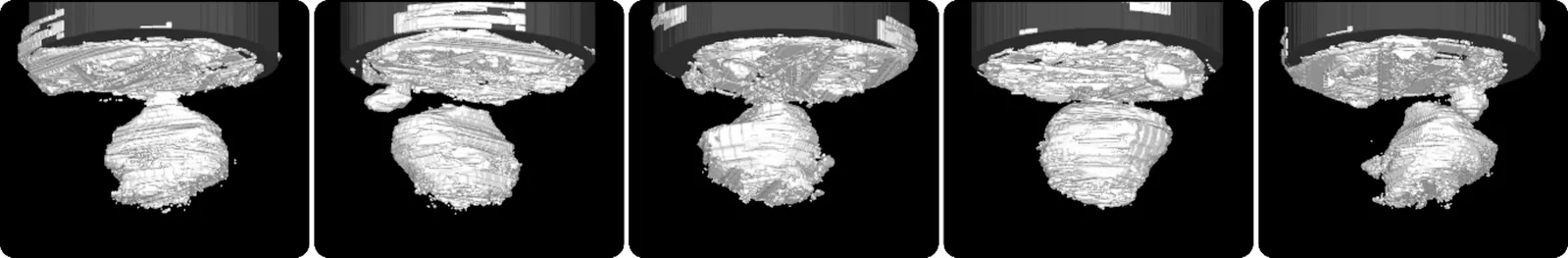
Five different views of 3D reconstructed cavitation cloud under the sonotrode tip. Animated sequences covering 360° field of view can be found in supplementary Video S3.

Following the qualitative inspection of the cavitation cloud images shown in Fig. 9, Fig. 10, ten additional experimental runs at horn amplitude of 50% were. Following the qualitative inspection of the cavitation cloud images shown in Fig. 9, Fig. 10, ten additional experimental runs at horn amplitude of 50% were performed. Together with the 75% amplitude case presented in Fig. 9, Fig. 10, these ten additional experiments resulted in a total of eleven datasets used for the quantitative analysis. Volume estimates from the 3D reconstructions using the SfS were compared with volumes estimated using the volume of revolution approach, where each half of the image was rotated by 180° − best possible volume estimation for a single image acquisition. A visual comparison between the original image (a) and the two estimation methods − (b) for volume of revolution and (c) for SfS reconstruction, respectively − used in the analysis is presented in Fig. 11.

**Fig. 11:**
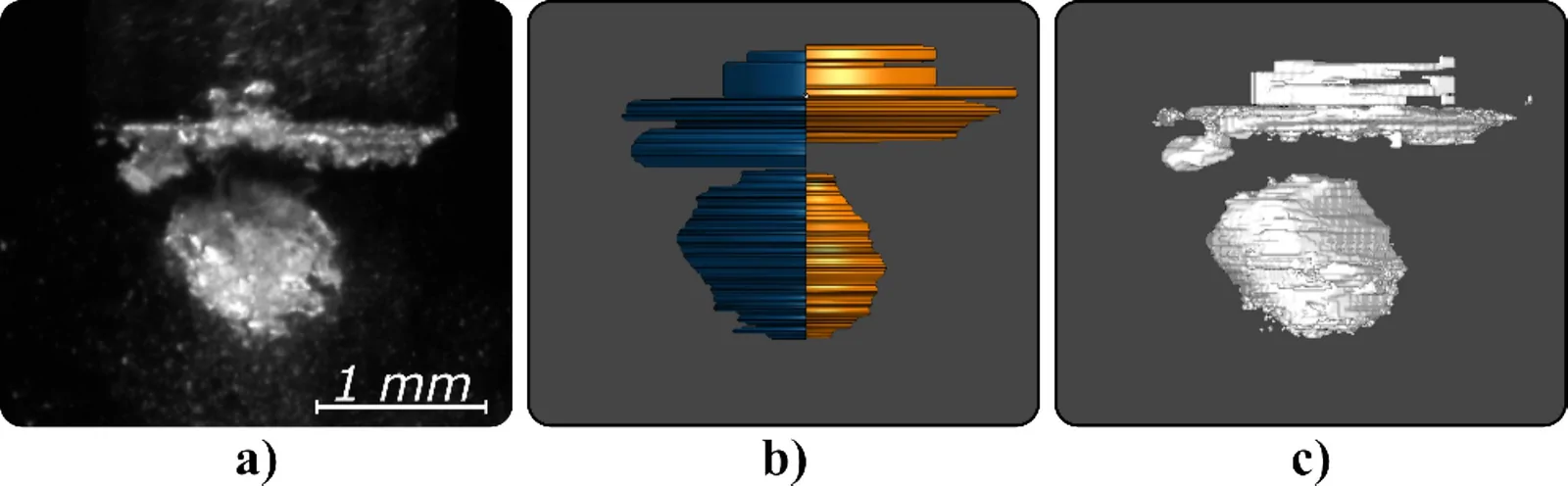
Visual comparison between a) input image as seen by one of the system cameras, b) volume of revolution and c) SfS reconstruction method. Animated sequences corresponding to a) and c) are provided as supplementary Video S3.

A quantitative comparison between the cavitation cloud volumes estimated using the volume of revolution approach and those obtained using the SfS reconstruction method is shown in Fig. 12. For every case, the volume measured from the SfS 3D reconstruction is compared to the volumes of revolution obtained from of each of the 30 viewpoints.

**Fig. 12:**
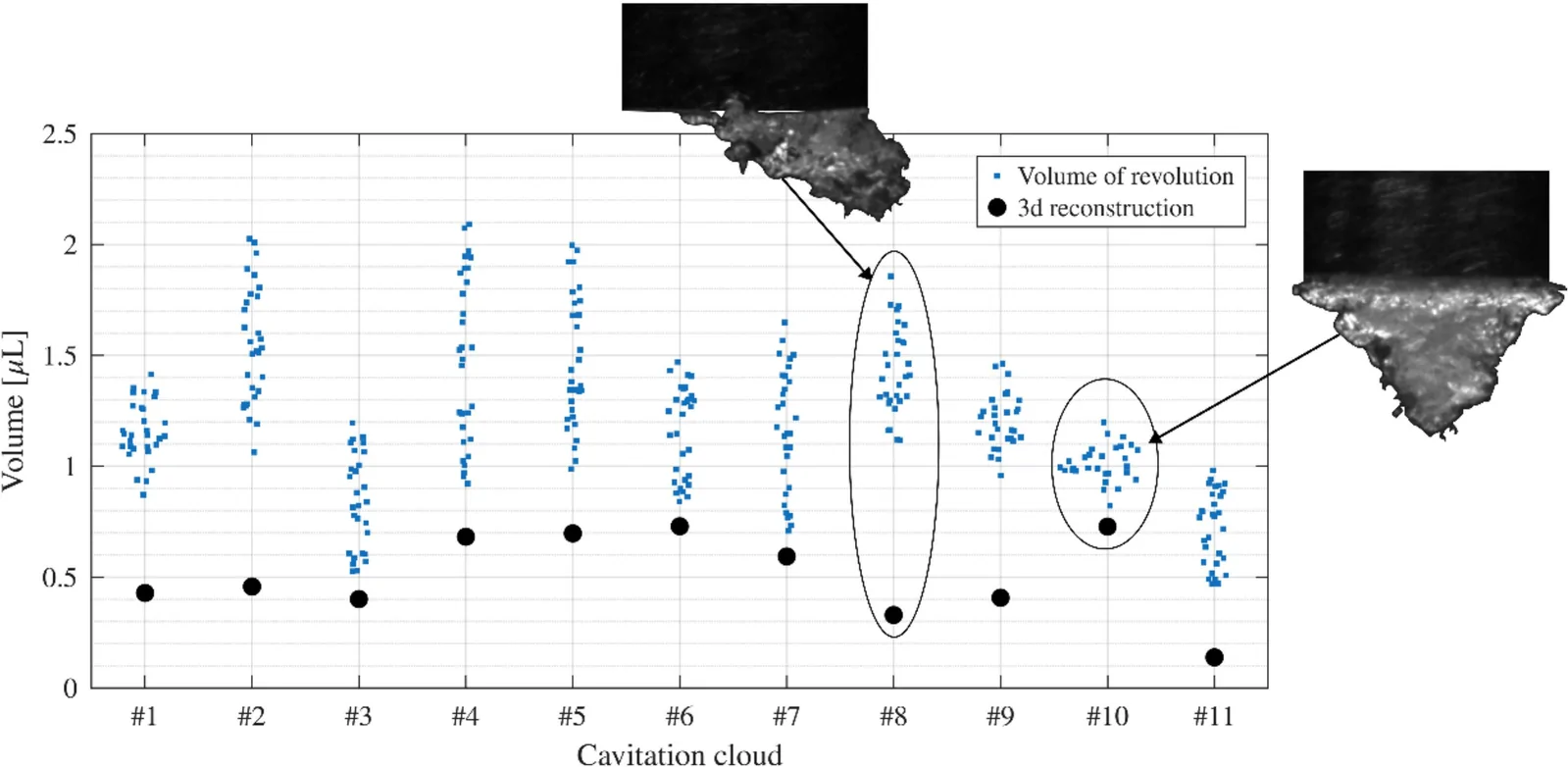
Comparison between cavitation cloud volume estimates obtained using the single view volume of revolution method and 30-viewpoint 3D SfS reconstruction. Individual observations are horizontally offset within each amplitude group to reduce marker overlap; the horizontal offset has no physical meaning. Animated sequences corresponding to cases #8 and #10 are provided in the Supplementary Material as Videos S4 and S5, respectively, and are strongly recommended for viewing, as they clearly illustrate the differences discussed in this section.

For all investigated cavitation clouds, the volume of revolution method yielded larger volume estimates than the SfS based 3D reconstructions. This indicates a systematic discrepancy between the two approaches, where the axisymmetric assumption inherent to the volume of revolution method leads to an overestimation of the cloud volume. As shown in the beeswarm plot in Fig. 12, some datasets exhibit smaller differences between the SfS reconstructed volume and the corresponding volume of revolution estimates. This behaviour is associated with the actual degree of asymmetry of the recorded cavitation cloud. More axisymmetric cavitation clouds tend to produce closer agreement between the SfS and volume of revolution estimates and, perhaps even more importantly, exhibit lower variability among the volume of revolution estimates. The magnitude of this discrepancy varied considerably among the investigated cases. The smallest discrepancy was observed for cavitation cloud #10, for which the volume of revolutions estimate exceeded the SfS reconstruction by 13%. In contrast, the largest discrepancy occurred for cavitation cloud #11, where the volume of revolution method produced up to a 7.1 times larger estimate. Across all 11 cavitation clouds, the average discrepancy amounted to +172%, confirming the systematic overestimation introduced by the axisymmetric volume of revolution assumption.

Fig. 13 presents the normalized volume estimates obtained for all 11 datasets described above. For each dataset, the estimates corresponding to one viewpoint were calculated using the single viewpoint volume of revolution method. The estimates corresponding to 3, 5, 6, 10, 15, and 30 viewpoints were obtained using the SfS reconstruction method with the respective numbers of camera views. The results show that the estimated volumes decrease asymptotically as the number of cameras used for reconstruction increases. Consistent with the tests performed using the physical reference object and the visualization system in Section 3, the range of volume estimates also decreases with an increasing number of cameras. This demonstrates that the system performs as intended under real experimental conditions. The same result also highlights the uncertainty of cavitation cloud volume estimates reported in previous studies when based solely on volume of revolution methods [37], [44].

**Fig. 13:**
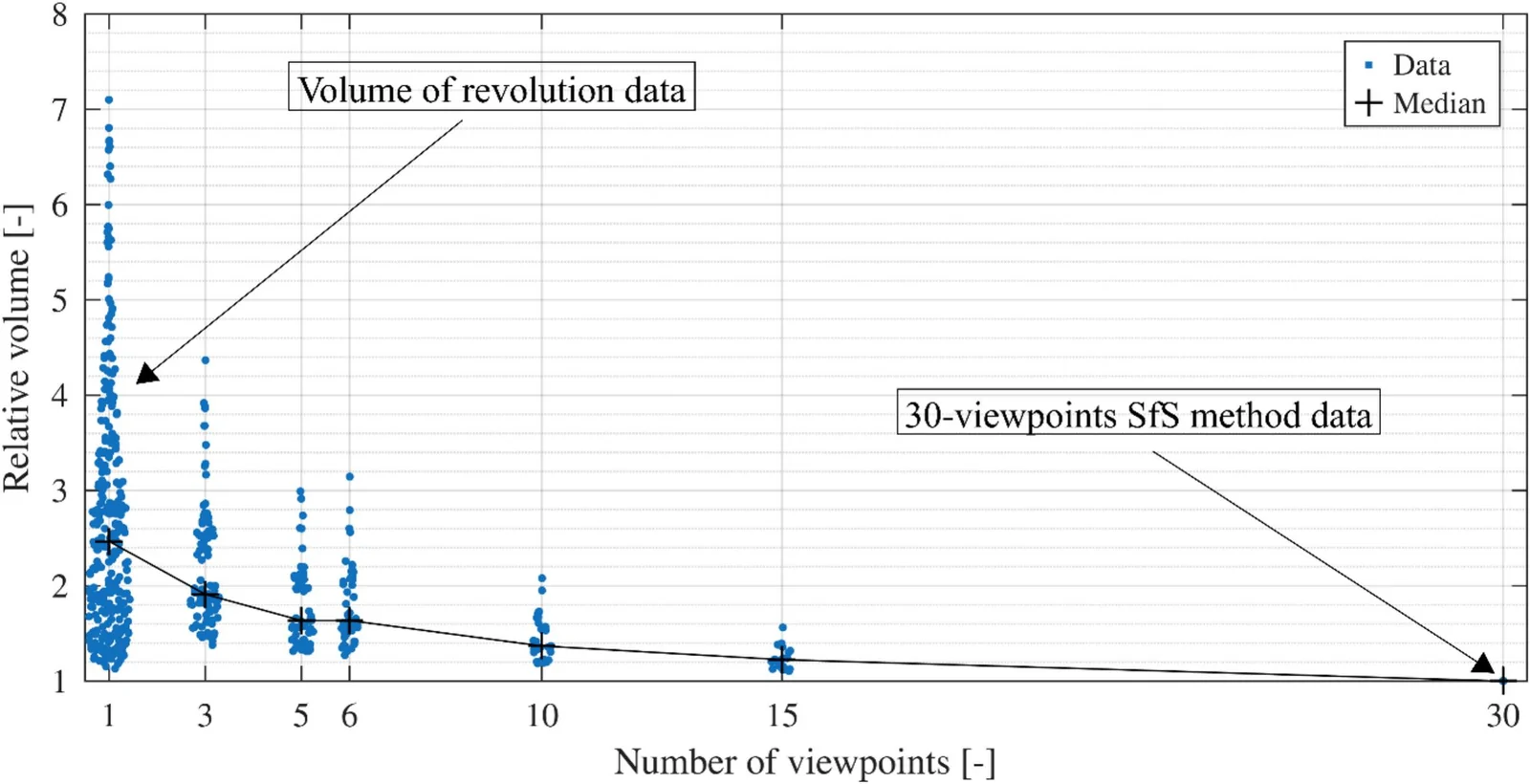
Normalized volume estimates for all data points obtained from the eleven datasets. For each image set, reconstructions were performed using all possible combinations of camera subsets corresponding to a given number of viewpoints. The 1 viewpoint results correspond to volume of revolution estimates, while the 3–30 viewpoint results correspond to SfS reconstructions. Individual observations are horizontally offset within each amplitude group to reduce marker overlap; the horizontal offset has no physical meaning.

The importance of multiple viewpoint observation can therefore be emphasized from two perspectives. First, volume estimates obtained from methods relying on a single observation direction are substantially more uncertain and can range from overestimated values to unrealistically underestimated ones. Second, qualitative evaluation of cavitation and other multiphase flow phenomena from a single viewpoint may lead to incorrect identification of morphological features or to their complete omission due to obstruction by other structures. Consequently, interpretations, classifications, or terminology based on similarities to 2D geometries or common organic shapes should be used with considerable caution or avoided where possible. To further support these observations, the reader is again referred to the supplementary Videos S3, S4 and S5.

### 5.2 Sequential image acquisition

With a constant delay Δt = 15 μs between the sequential camera triggers, the effect of a single conventional highspeed camera that circles around phenomenon at a constant radius with a constant velocity and records at a frame rate of 66,666 fps was recreated (Fig. 14).

**Fig. 14:**
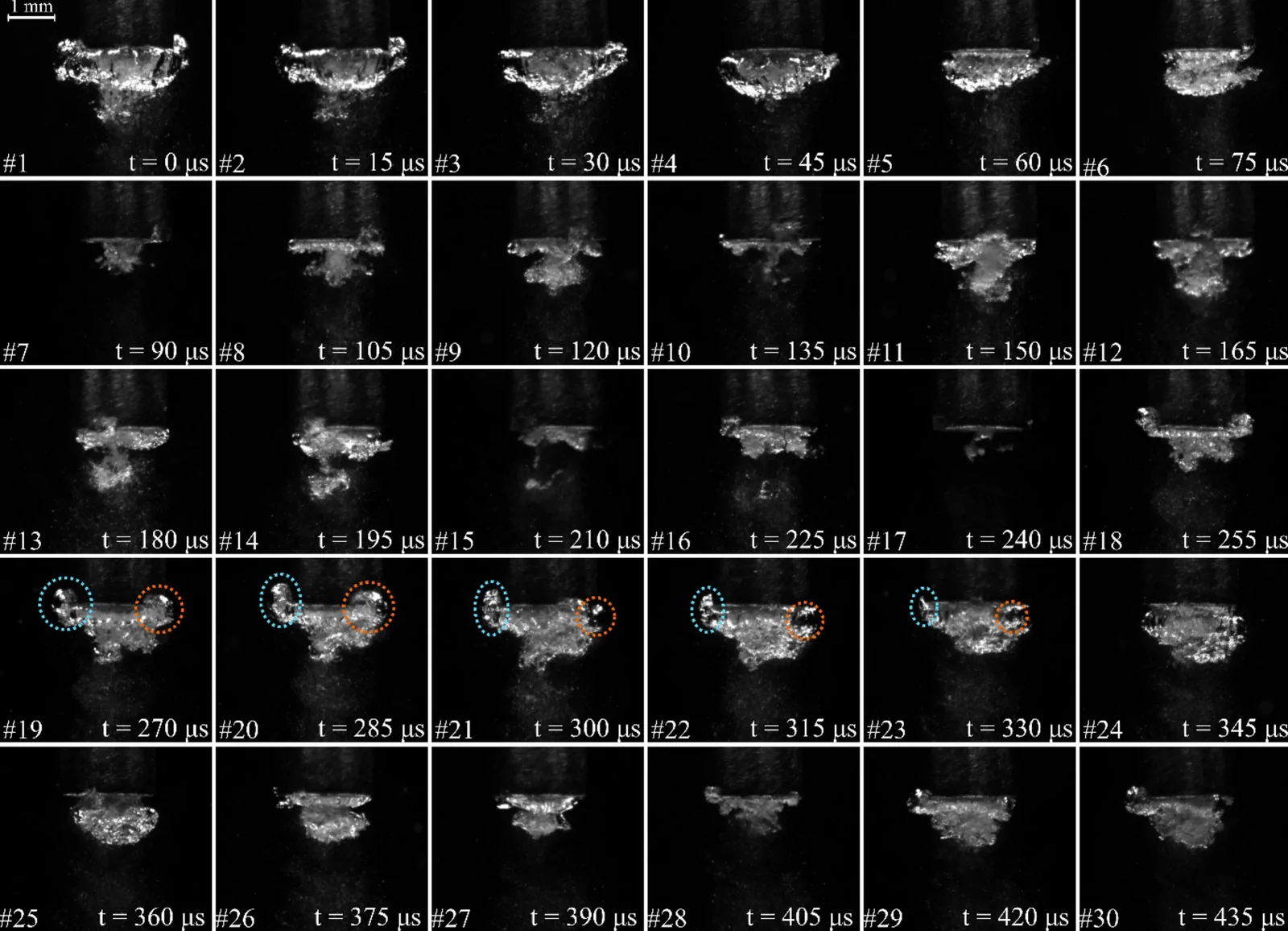
Sequence of 30 images recorded at Δt = 15 μs, showing the evolution of cavitation dynamics at the ultrasonic horn tip over approximately two cavitation oscillation cycles. Additional animated views are provided in supplementary Video S6.

The sequence captures the dynamics of a cavitation cloud at the sonotrode tip over a total duration of t_tot_ = 435 μs. This recording interval corresponds to slightly more than two complete oscillation cycles of the cavitation cloud. Previous studies have shown that the characteristic oscillation frequency of cavitation clouds generated at ultrasonic horns with small tips typically lies in the range of 3–5 kHz [34].

The image sequence reveals a rich morphological composition of the cavitation cloud throughout its evolution, from growth to collapse. Several distinct structures remain visible across consecutive frames. For example, the growth and subsequent collapse of two bubble-like structures can be observed between timestamps #19 and #23, highlighted by blue and orange dashed ellipses. Because the fields of view of neighbouring cameras partially overlap, such persistent features can be followed over time and from changing viewing angles. As in the snapshot mode observations, the asymmetric nature of the cavitation cloud is clearly evident throughout the sequence. A 3D reconstruction was not performed in this specific case, as the example is intended solely to illustrate a potential direction for future methodological development and research.

It should again be emphasized that the cavitation cloud dynamics and associated structural features shown in Fig. 14 can be observed and interpreted more clearly in the animated sequences provided in the supplementary material (Video S6).

### 5.3 Statistical analysis of cavitation cloud volume and spatial distribution

Statistical characterization based on time averaged or ensemble averaged quantities is commonly used in ultrasonic cavitation research to describe inherently transient and highly variable phenomena. In particular, single viewpoint image derived parameters such as the projected cavitation cloud area or summed bubble area are frequently evaluated over large numbers of image frames to characterize the mean extent and temporal behaviour of cavitation structures [36], [45]. Building on this approach, the post processed data obtained with the present multiple viewpoint visualization system were subjected to a statistical analysis. The objective was to investigate properties that cannot be reliably assessed from conventional 2D observations, particularly the occurrence, persistence, and spatial distribution of cavitation clouds and their asymmetries. More specifically, multiple “snapshot mode” datasets were acquired at different displacement amplitudes and subsequently reconstructed in 3D. The reconstructions were then used to estimate the cavitation cloud volume and to characterize the general 3D shape of the cloud across the investigated operating conditions.

An extensive experimental campaign was performed in snapshot mode, with 100 simultaneous image sets acquired at each sonotrode amplitude setting between 20% and 100% in increments of 10%. The corresponding cavitation cloud volumes were obtained from the 3D reconstructions. Fig. 15 presents the individual volume estimates together with the mean value and the 95th percentile, the latter used here as a statistical indicator of the upper range of observed cavitation cloud volumes.

**Fig. 15:**
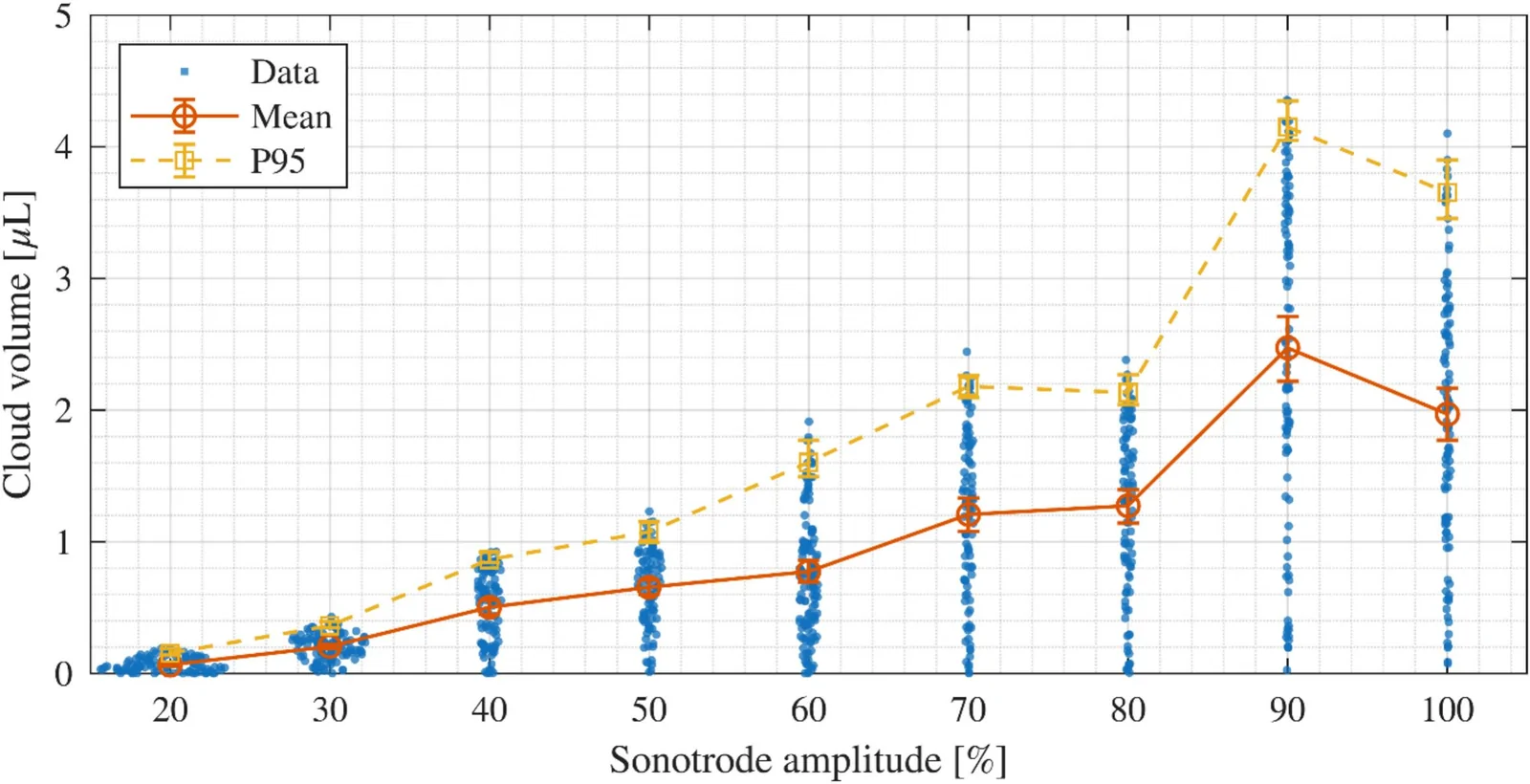
Cavitation cloud volume as a function of sonotrode amplitude based on 100 snapshot mode reconstructions at each amplitude setting. The solid line shows the mean volume, while the second line represents the 95th percentile. Individual observations are horizontally offset within each amplitude group to reduce marker overlap; the horizontal offset has no physical meaning. Error bars represent the 95% confidence intervals of the corresponding metric estimates.

The results show a general increase in cavitation cloud volume with increasing sonotrode amplitude up to 90%, where both the mean and the 95th percentile reach a maximum. At 100% amplitude, both quantities decrease, indicating that the relationship between cavitation cloud volume and driving amplitude is not strictly monotonic over the investigated range. The similar evolution of the mean and 95th-percentile curves suggests that this behaviour is not limited to isolated realizations but reflects a broader change in the volumetric response of the cavitation cloud.

The decrease in cavitation cloud volume observed between 90% and 100% amplitude suggests a non-monotonic response at the highest excitation levels. Although directly comparable volumetric measurements are scarce, similar changes in cavitation behaviour at high sonotrode amplitudes have been reported by Khavari et al. [46], who observed substantial modification of the cavitation field and reduced acoustic pressure beneath the horn at higher input powers due to cavitation shielding by the bubble cloud. A related non-monotonic response was also reported by Yusuf et al. [45], who observed reduced shock-wave activity at transitional sonotrode vibration amplitudes despite increasing input power.

To further characterize the spatial distribution of the cavitation cloud, the 100 3D reconstructions obtained at each sonotrode amplitude were combined into a voxel occupancy map. All reconstructions were positioned within a common coordinate system referenced to the sonotrode tip. For each reconstruction, voxels occupied by the reconstructed cavitation cloud contributed a value of one to the corresponding position in the common voxel domain. The accumulated values were subsequently divided by the total number of reconstructions, resulting in a local voxel occupancy expressed as a percentage. The resulting 3D field therefore represents how frequently each spatial location was occupied by the cavitation cloud over the 100 recorded realizations.

Representative cross sections of the resulting occupancy fields are shown in Fig. 16. The rows correspond to different tip displacement amplitudes, while the columns represent transverse slices at increasing axial distances from the sonotrode tip. The rightmost column shows a slice through the center of the horn at y = 0. The colour scale indicates the percentage of reconstructions in which a given voxel was occupied. Consequently, regions of high occupancy represent spatial locations repeatedly occupied by the cavitation cloud, whereas regions of low occupancy correspond to less frequently occurring structures. The mesh squares are 500 × 500 μm in size.

**Fig. 16:**
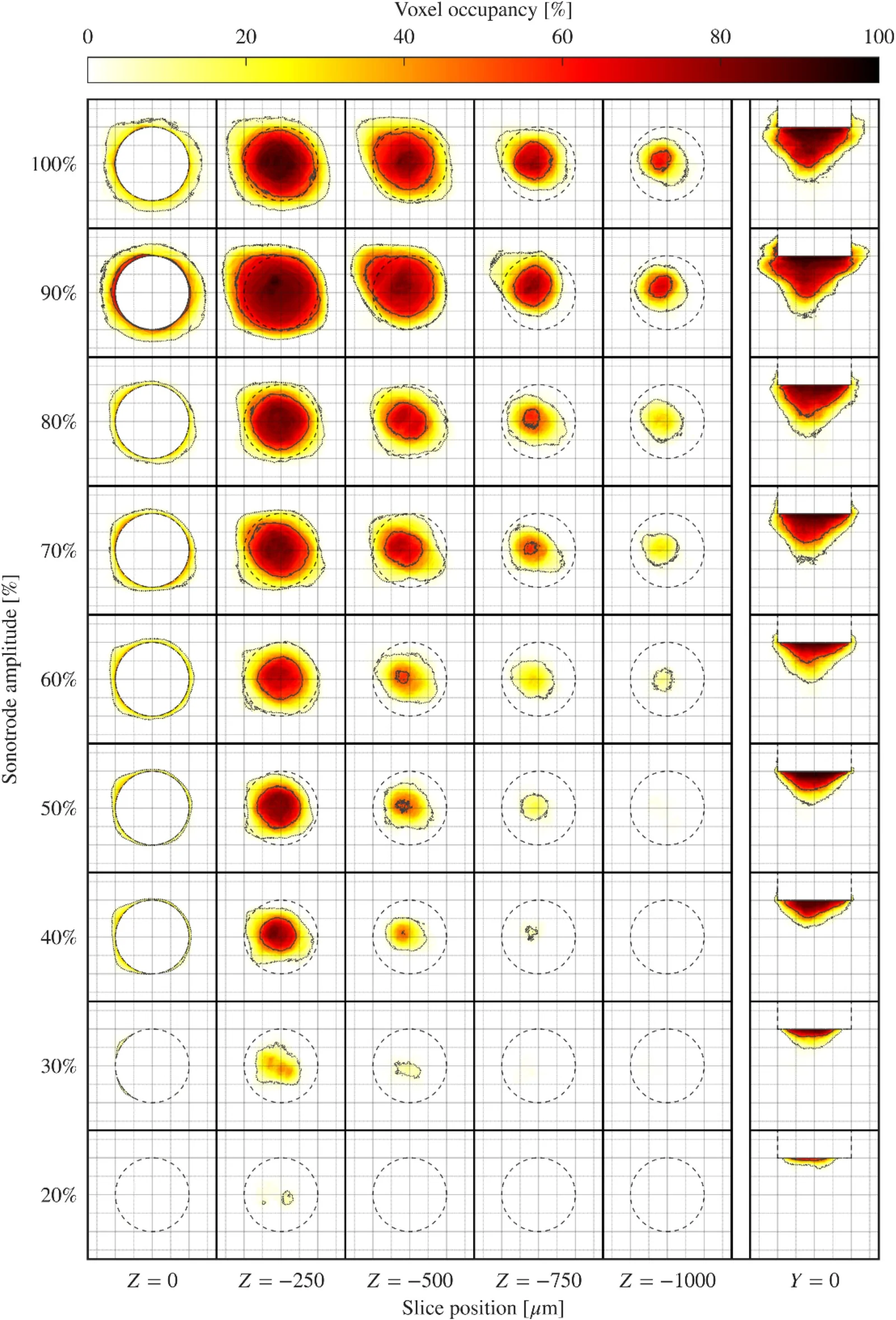
Cross sections of the averaged 3D voxel occupancy for sonotrode amplitude settings from 20% to 100%. The first five columns show transverse slices at different axial positions Z below the sonotrode tip, while the last column shows the central longitudinal slice at Y = 0. An extended representation containing longitudinal slices at different Y positions is provided in the Supplementary Material.

The averaged voxel occupancy maps show that the spatial distribution of the cavitation cloud does not become fully axisymmetric, even when based on 100 independent realizations. Deviations from circular symmetry remain visible, particularly at higher sonotrode amplitudes, indicating that the observed asymmetry is not solely the result of randomly oriented transient structures. Instead, certain spatial directions appear to be preferentially occupied across repeated realizations. The occupancy distribution also suggests a distinction between a more repeatable inner region and a more variable outer region of the cavitation field. The latter may represent a less stable part of the flow in which asymmetric structures are more likely to develop, although this interpretation remains qualitative. As an additional measure of asymmetry, the centroids of the reconstructed cavitation clouds were also analysed. A statistically significant displacement of the centroids from the sonotrode axis was observed, supporting the voxel occupancy results and indicating that the asymmetries do not completely average out across repeated realizations. Error bars represent the 95% confidence intervals of the corresponding metric estimates. The corresponding analysis is provided in the Supplementary Material.

The physical origin of these preferred asymmetries cannot be determined from the present measurements alone. The present results therefore only demonstrate that the cavitation field can retain a statistically preferred non-axisymmetric structure despite the nominally axisymmetric geometry of the sonotrode.

## 6 Conclusions

This study demonstrated the feasibility of a multiple viewpoint visualization system for investigating small scale multiphase flows, with particular emphasis on cavitation. The developed 30-camera system provided simultaneous circumferential observations of cavitation structures at length scales as small as 2 mm and enabled both qualitative analysis and approximate 3D volume estimation. Two acquisition modes were demonstrated: snapshot mode for simultaneous spatial observation and virtual spinning highspeed camera mode for sequential observation of cavitation dynamics.

The snapshot mode results demonstrated the value of observing cavitation from multiple directions. Structures that appeared symmetric or continuous from a single viewpoint were frequently revealed to be strongly asymmetric or composed of several overlapping features when viewed from other directions. Statistical analysis of repeated 3D reconstructions further showed that these asymmetries do not completely average out across multiple realizations. Preferred non-axisymmetric spatial distributions remained visible in the averaged voxel occupancy fields, particularly at higher sonotrode amplitudes, despite the nominally axisymmetric geometry of the sonotrode.

The results also demonstrated the limitations of estimating cavitation cloud volume from a single viewpoint under an assumption of axisymmetry. Compared with the volume of revolution approach, the multiple viewpoint SfS method provided more consistent volume estimates, while the reconstruction tests confirmed that accuracy and repeatability improve as the number of viewpoints increases. Repeated measurements at sonotrode amplitudes between 20% and 100% additionally showed a general increase in cavitation cloud volume with increasing amplitude, followed by a decrease between 90% and 100%, indicating that the volumetric response is not strictly monotonic at the highest excitation levels.

Future work will focus on improving the optical quality and reconstruction methodology to achieve more accurate volume estimation and more detailed geometrical reconstruction. Further development could also enable investigation of the temporal behaviour and physical origin of the statistically preferred asymmetries identified in the present study. Extension of the method to hydrodynamic cavitation is also foreseen, particularly for applications in which local 3D flow structures are important, such as cavitation erosion. The system may additionally provide useful experimental data for validation of 3D CFD simulations of multiphase and cavitating flows.

Although the current system cannot resolve micron scale features and the applied reconstruction procedure remains simplified, the results demonstrate its potential for both qualitative and quantitative analysis of complex multiphase phenomena. With further refinement, the proposed system can provide a versatile platform for spatially resolved experimental investigation of cavitating and other multiphase flows.

## CRediT authorship contribution statement

**Jernej Ortar:** Writing – original draft, Visualization, Methodology, Investigation, Data curation. **Jure Zevnik:** Writing – original draft, Visualization, Methodology, Data curation. **Matej Sečnik:** Methodology. **Marko Hočevar:** Supervision, Conceptualization. **Matevž Dular:** Writing – original draft, Supervision, Methodology, Conceptualization.

## Declaration of Generative AI and AI-assisted technologies in the writing process

During the preparation of this work the authors used ChatGPT only to improve language and readability. After using this tool, the authors reviewed and edited the content as needed and take full responsibility for the content of the publication.

## Declaration of competing interest

The authors declare that they have no known competing financial interests or personal relationships that could have appeared to influence the work reported in this paper.

## Appendix A Supplementary data

Supplementary Fig. 1 Extended cross sections of the 3D voxel occupancy for sonotrode amplitude settings from 20% to 100%, associated with Fig. 16. The columns show longitudinal slices at different positions. The colour scale indicates the percentage of the 100 reconstructed cavitation clouds occupying each voxel position.

Supplementary Fig. 2 Mean radial and vertical displacement of the reconstructed cavitation cloud centroids as a function of sonotrode amplitude. Error bars represent the 95% confidence intervals of the corresponding metric estimates.

Supplementary video 1 Full 360° view of the CAD test object (left) and its 3D reconstruction (right) associated with Fig. 6a).

Supplementary video 2 Full 360° view of the 3D printed test object (left) and its 3D reconstruction (right) associated with Fig. 6b).

Supplementary video 3 Full 360° view of cavitation cloud as observed by the 30 cameras (left) and its 3D reconstruction (right) associated with Fig. 9.

Supplementary video 4 Full 360° view of cavitation cloud as observed by the 30 cameras of the multipleviewpoint visualization system associated with Fig. 12 - #8.

Supplementary video 5 Full 360° view of cavitation cloud as observed by the 30 cameras of the multipleviewpoint visualization system associated with Fig. 12 - #10.

Supplementary video 6 Full 360° view of the cavitation cloud as observed by the 30-camera system with a 15 µs delay between successive camera recordings, associated with Fig. 14.

## References

[1] Villafañe L., Aliseda A., Ceccio S., Di Marco P., Machicoane N., Heindel T.J. (2025). 50 Years of International Journal of Multiphase Flow: experimental methods for dispersed multiphase flows. Int. J. Multiph. Flow.

[2] Yeoh G.H. (2019). Thermal hydraulic considerations of nuclear reactor systems: past, present and future challenges. Exp. Comput. Multiph. Flow.

[3] Poelma C. (2020). Measurement in opaque flows: a review of measurement techniques for dispersed multiphase flows. Acta Mech..

[4] Garcia-Villalba M., Colonius T., Desjardins O., Lucas D., Mani A., Marchisio D., Matar O.K., Picano F., Zaleski S. (2025). Numerical methods for multiphase flows. Int. J. Multiph. Flow.

[5] Subramaniam S. (2020). Multiphase flows: Rich physics, challenging theory, and big simulations. Phys. Rev. Fluids.

[6] Jahangir S., Wagner E.C., Mudde R.F., Poelma C. (2019). Void fraction measurements in partial cavitation regimes by X-ray computed tomography. Int. J. Multiph. Flow.

[7] Tekawade A., Sforzo B., Matusik K., Fezzaa K., Kastengren A., Powell C. (2020). Time-resolved 3D imaging of two-phase fluid flow inside a steel fuel injector using synchrotron X-ray tomography. Sci. Rep..

[8] Mitroglou N., Lorenzi M., Santini M., Gavaises M. (2016). Application of X-ray micro-computed tomography on high-speed cavitating diesel fuel flows. Exp. Fluids.

[9] Pleterski Ž., Hočevar M., Bizjan B., Kolbl Repinc S., Rak G. (2023). Measurements of complex free water surface topography using a photogrammetric method. Remote Sens. (Basel).

[10] Alberini F., Liu L., Stitt E.H., Simmons M.J.H. (2017). Comparison between 3-D-PTV and 2-D-PIV for determination of hydrodynamics of complex fluids in a stirred vessel. Chem. Eng. Sci..

[11] Foeth E.J., van Doorne C.W.H., van Terwisga T., Wieneke B. (2006). Time resolved PIV and flow visualization of 3D sheet cavitation. Exp. Fluids.

[12] A. Kleinwächter, K. Hellwig-Rieck, E. Ebert, R. Kostbade, H.-J. Heinke, N. Damaschke, PIV as a novel full-scale measurement technique in cavitation research, 2015.

[13] Dular M., Bachert R., Schaad C., Stoffel B. (2007). Investigation of a re-entrant jet reflection at an inclined cavity closure line. Eur. J. Mech. B-Fluids.

[14] Lauterborn W., Hentschel W. (1986). Cavitation bubble dynamics studied by high speed photography and holography: part two. Ultrasonics.

[15] Appel J., Koch P., Mettin R., Krefting D., Lauterborn W. (2004). Stereoscopic high-speed recording of bubble filaments. Ultrason. Sonochem..

[16] Prédiction de l’érosion de cavitation: approche énergétique, EPFL, 1997. doi:10.5075/epfl-thesis-1592.

[17] Pereira F.J.A., Avellan F., Dupont P. (1998). Prediction of cavitation erosion: an energy approach. J. Fluids Eng..

[18] Szeliski R. (2022).

[19] Sonka M., Hlavac V., Boyle R. (2014).

[20] E. Ebert, N. Damaschke, Synchronized Multi-camera and LED-illumination system for multi perspective cavitation observation and 3D reconstruction, in: Rome, 2019.

[21] L. Savio, M. Viviani, F. Conti, M. Ferrando, Application of computer vision techniques to measure cavitation bubble volume and cavitating tip vortex diameter., (2009). http://deepblue.lib.umich.edu/handle/2027.42/84235 (accessed June 14, 2024).

[22] Franzosi G., Abbasi A.A., Savio L., Viviani M., Ferrando M., Tani G. (2025). Experimental study of bubble cavitation on a NACA 0015 hydrofoil by a computer vision approach. Ocean Eng..

[23] Yan W. (2023). 4th International Seminar on Artificial Intelligence, Networking and Information Technology (AINIT).

[24] K. Shiraishi, Y. Sawada, D. Arakawa, 3D Shape Measurement Method for Cavitation on Marine Propellers Using Combination Line CCD Camera Method -Experimental Estimation for Pressure Fluctuation on Ship Stern Induced by Cavitating Propeller Using Measurement Results of Cavitation Volume- - openasfa.title, (n.d.). https://www.fao.org/fishery/en/openasfa/c870562c-b0e5-4a93-945b-a4f9d9124c3c (accessed June 14, 2024).

[25] Saito M., Kanai T. (2024). Development of multi-view 3D reconstruction system for bubble flow measurement. Flow Meas. Instrum..

[26] Fu Y., Liu Y. (2018). 3D bubble reconstruction using multiple cameras and space carving method. Meas. Sci. Technol..

[27] Masuk A.U.M., Salibindla A., Ni R. (2019). A robust virtual-camera 3D shape reconstruction of deforming bubbles/droplets with additional physical constraints. Int. J. Multiph. Flow.

[28] Choi H.M., Terauchi T., Monji H., Matsui G. (2002). Visualization of bubble-fluid interaction by a moving object flow image analyzer system. Ann. N. Y. Acad. Sci..

[29] Li M., Sankin G., Vu T., Yao J., Zhong P. (2021). Tri-modality cavitation mapping in shock wave lithotripsy. J. Acoust. Soc. Am..

[30] Wang H., Xu H., Pooneeth V., Gao X.-Z. (2018). A novel one-camera-five-mirror three-dimensional imaging method for reconstructing the cavitation bubble cluster in a water hydraulic valve. Appl. Sci..

[31] Wang H., Xu H., Gao X.-Z., Zhao Z., Huang J. (2021). Arrangement optimization of a novel three dimensional multiphase flow imaging device employing modified harmony search algorithm. Eng. Appl. Artif. Intel..

[32] Kim K.-H., Chahine G., Franc J.-P., Karimi A. (2014). Advanced Experimental and Numerical Techniques for Cavitation Erosion Prediction.

[33] Dular M., Petkovšek M. (2015). On the mechanisms of cavitation erosion – Coupling high speed videos to damage patterns. Exp. Therm. Fluid Sci..

[34] Žnidarčič A., Mettin R., Dular M. (2015). Modeling cavitation in a rapidly changing pressure field – application to a small ultrasonic horn. Ultrason. Sonochem..

[35] Al Bishtawi B., Mustapha K.B., Scribano G. (2024). Molding the acoustic cavity–analyzing the influence of toroidal vortex development on acoustic multi-bubble macrostructures under different ultrasonic horn tip diameters. Phys. Fluids.

[36] Kozmus G., Zevnik J., Hočevar M., Dular M., Petkovšek M. (2022). Characterization of cavitation under ultrasonic horn tip – proposition of an acoustic cavitation parameter. Ultrason. Sonochem..

[37] Mottyll S., Skoda R. (2016). Numerical 3D flow simulation of ultrasonic horns with attached cavitation structures and assessment of flow aggressiveness and cavitation erosion sensitive wall zones. Ultrason. Sonochem..

[38] Ma X., Huang B., Wang G., Zhang M. (2017). Experimental investigation of conical bubble structure and acoustic flow structure in ultrasonic field. Ultrason. Sonochem..

[39] Yasui K., Iida Y., Tuziuti T., Kozuka T., Towata A. (2008). Strongly interacting bubbles under an ultrasonic horn. Phys. Rev. E.

[40] Bai L., Xu W., Deng J., Li C., Xu D., Gao Y. (2014). Generation and control of acoustic cavitation structure. Ultrason. Sonochem..

[41] Schreiner F., Gianfelice T., Skoda R. (2024). Three-dimensional simulation of an orifice flow with cavitation-induced air release. Int. J. Multiph. Flow.

[42] Laurentini A. (1994). The visual hull concept for silhouette-based image understanding. IEEE Trans. Pattern Anal. Machine Intell..

[43] Hartley R., Zisserman A. (2004).

[44] Žnidarčič A., Mettin R., Cairós C., Dular M. (2014). Attached cavitation at a small diameter ultrasonic horn tip. Phys. Fluids.

[45] Yusuf L., Symes M.D., Prentice P. (2021). Characterising the cavitation activity generated by an ultrasonic horn at varying tip-vibration amplitudes. Ultrason. Sonochem..

[46] Khavari M., Priyadarshi A., Hurrell A., Pericleous K., Eskin D., Tzanakis I. (2021). Characterization of shock waves in power ultrasound. J. Fluid Mech..

